
JOURNAL INFORMATION
==============================
NLM Title Abbreviation: Biospektrum (Heidelb)
Journal ID: Biospektrum (Heidelb)
NLM Journal ID: 9615685

Biospektrum

ISSN: 0947-0867
EISSN: 1868-6249

ARTICLE INFORMATION
==============================
PMCID: PMC8417673
PMID: 34511734
DOI: 10.1007/s12268-021-1620-6
Article ID: 1620
Article version: 1
Subjects: Wissenschaft

MicroRNAs bei COVID-19: kleine Moleküle mit großem Potenzial?

Bär Christian 1 2
Derda Anselm A. 1 3
Thum Thomas thum.thomas@mh-hannover.de
1 2 4
1 grid.10423.34 0000 0000 9529 9877 Institut für Molekulare und Translationale Therapiestrategien, Medizinische Hochschule Hannover, Carl-Neuberg-Str. 1, D-30625 Hannover, Deutschland
2 grid.10423.34 0000 0000 9529 9877 Rebirth - Forschungszentrum für Translationale Regenerative Medizin, Medizinische Hochschule Hannover, Hannover, Deutschland
3 grid.10423.34 0000 0000 9529 9877 Klinik für Kardiologie und Angiologie, Medizinische Hochschule Hannover, Hannover, Deutschland
4 grid.418009.4 0000 0000 9191 9864 Fraunhofer-Institut für Toxikologie und Experimentelle Medizin, Hannover, Deutschland
Electronic publication date: 2021 Sep 4
Print publication date: 2021
Volume: 27
Issue: 5
First page: 485
Last page: 487
Copyright: © Die Autoren 2021
License: Dieser Artikel wird unter der Creative Commons Namensnennung 4.0 International Lizenz veröffentlicht, welche die Nutzung, Vervielfältigung, Bearbeitung, Verbreitung und Wiedergabe in jeglichem Medium und Format erlaubt, sofern Sie den/die ursprünglichen Autor(en) und die Quelle ordnungsgemäß nennen, einen Link zur Creative Commons Lizenz beifügen und angeben, ob Änderungen vorgenommen wurden. Die in diesem Artikel enthaltenen Bilder und sonstiges Drittmaterial unterliegen ebenfalls der genannten Creative Commons Lizenz, sofern sich aus der Abbildungslegende nichts anderes ergibt. Sofern das betreffende Material nicht unter der genannten Creative Commons Lizenz steht und die betreffende Handlung nicht nach gesetzlichen Vorschriften erlaubt ist, ist für die oben aufgeführten Weiterverwendungen des Materials die Einwilligung des jeweiligen Rechteinhabers einzuholen. Weitere Details zur Lizenz entnehmen Sie bitte der Lizenzinformation auf http://creativecommons.org/licenses/by/4.0/deed.de.
License URL: https://creativecommons.org/licenses/by/4.0/

issue-copyright-statement: © Springer-Verlag GmbH Deutschland, ein Teil von Springer Nature 2021

==============================
COVID-19 still remains a severe global health threat. Despite the high-speed development of vaccines that efficiently prevent COVID-19, there are still no effective treatments of the disease once people are infected. MicroRNAs are powerful regulators of gene expression. They are intensely investigated as therapeutic targets up to the clinical stage. In addition, microRNAs can be detected in the circulation, and thus, represent promising diagnostic and prognostic biomarkers for (long)-COVID-19.

■ Die COVID-19-Pandemie hat auch fast eineinhalb Jahre nach dem ersten Auftreten die Welt fest im Griff und hat nach offiziellen Zahlen der Weltgesundheitsorganisation bereits mehr als vier Millionen Menschleben gefordert. Neben den primären Manifestationen, wie Lungenentzündung und akutem Lungenversagen (*acute respiratory distress syndrome*, ARDS), kommt es häufig zu einem Multiorganversagen, da durch den Erreger, das SARS-CoV-2, neben der Lunge auch andere Organe befallen werden. Sehr häufig kommt es zu einer kardialen Beteiligung – sowohl akut, aber auch chronisch. Wie eine deutsche Beobachtungsstudie kürzlich zeigte, wurden nach überstandener Infektion bei bis zu 60 Prozent der Patienten Herzmuskelentzündungen festgestellt [1]. Des Weiteren sind kardiovaskuläre Vorerkrankungen ein erheblicher Risikofaktor für schwere COVID-19 Verläufe [2]. Zurückzuführen ist dies wahrscheinlich auf das Angiotensin-konvertierendes Enzym 2 (ACE2), das als primärer Zelleintrittsrezeptor für SARS-CoV-2 dient und neben Alveolarepithelzellen auch stark auf der Oberfläche von Kardiomyozyten exprimiert ist. Während die Entwicklung neuartiger und höchst effektiver Vektor- und mRNA-Impfstoffe zur Verhinderung der Krankheit mit beeindruckendem Tempo voranschreitet, gab es bei der Behandlung von schwerkranken COVID-19-Infizierten bisher leider keinen Durchbruch. Unsere Arbeiten fokussieren sich vor allem auf die Erforschung von nicht codierenden RNAs als neuartige Zielstrukturen für die Behandlung, Diagnose und Prognose kardiovaskulärer Erkrankungen. Wir gehen davon aus, dass die nicht codierenden RNAs auch bei der Behandlung von COVID-19, vor allem bei den begleitenden kardialen Manifestationen, eine wichtige Rolle spielen können.

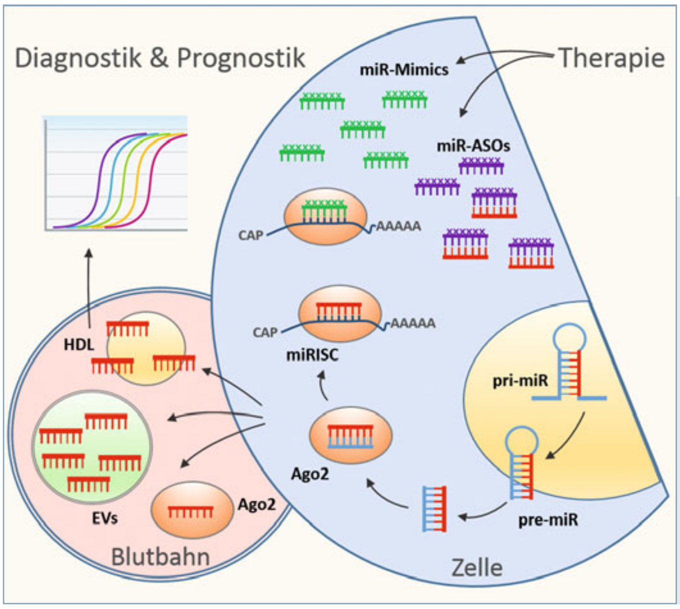

◀ **Abb. 1:** Schematische Darstellung der microRNA-Biosynthese. Die Synthese der microRNAs (miRs) findet im Zellkern statt. Die pri-miR-Vorläufer werden durch Nukleasen zu pre-miRs und schließlich der reifen doppelsträngigen miR prozessiert. Gebunden an Argonaut-Proteine bildet einer der beiden Stränge den *miRNA-induced silencing complex* (miRISC), der durch komplementäre Basenpaarung vermittelt messenger-RNAs destabilisiert oder deren Translation blockiert. Dieser Wirkmechanismus der microRNAs kann therapeutisch durch Einbringen synthetischer microRNAs (miR-Mimic) sowie Inhibitoren (*miRNA antisense oligonucleotides*, miR-ASO) manipuliert werden. Zudem können die microRNAs entweder verpackt in extrazelluläre Vesikel (EVs) oder gebunden an RNA-Bindeproteine wie das Argonaut2 (Ago2) oder *high-density*-Lipoproteine (HDL) ins Blut abgegeben werden und stellen daher vielversprechende diagnostische und prognostische Krankheitsbiomarker dar.

## Funktionsweise und therapeutisches Potenzial der microRNAs

MicroRNAs repräsentieren eine Unterklasse der sehr heterogenen Welt funktioneller, nicht codierender RNAs. Sie liegen im Cytoplasma von Zellen als einzelsträngige RNA-Moleküle vor, im Allgemeinen mit einer Länge von 20–22 Nukleotiden, und sind bei der posttrankriptionellen Regulation von zellulären Signalwegen von entscheidender Bedeutung. MicroRNAs werden durch Argonaut-Proteine gebunden und bilden den *miR-induced silencing complex* (miRISC), der durch komplementäre Basenpaarungen der microRNA an spezifische mRNAs bindet, was zur Hemmung der Proteintranslation oder zur Destabilisierung und Abbau der mRNA führt [3]. Dieses Prinzip kann man sich therapeutisch zunutze machen, indem man synthetische microRNAs (miR-Mimics) in Zellen einbringt, um deren Aktivität zu erhöhen. Alternativ kann man die Funktion der microRNAs durch das Einbringen von spezifischen Antisense-Oligonukleotiden (miR-ASO) blockieren (**Abb. 1**). Als Paradebeispiel dient die microRNA-132, welche durch erhöhte Expressionslevel unter kardialen Stressbedingungen (z. B. chronischem Bluthochdruck) maßgeblich zur Entstehung der kardialen Hypertrophie und Herzinsuffizienz beiträgt. Die Blockade der microRNA-132 mittels spezifischer Antisense-Oligonukleotide hat sich als effektive therapeutische Strategie im Klein- und Großtiermodell erwiesen und hat bereits in einer ersten klinischen Studie (Phase 1b) vielversprechende Ergebnisse bei der Behandlung von Herzinsuffizienzpatienten gezeigt [4, 5].

## MicroRNA-Therapie bei COVID-19?

Auf der Grundlage dieser Erkenntnisse stellte sich für uns die Frage, ob microRNAs auch zur Behandlung von COVID-19 dienen könnten. In unserer ersten Konzeptstudie fokussierten wir uns zunächst auf ACE2, das Schlüsselmolekül für den Zelleintritt von SARS-CoV-2. Wie zeitgleich auch aus anderen Studien bekannt wurde, konnten wir zeigen, dass bei den verschiedenen Zelltypen des Herzens ACE2 hauptsächlich in Kardiomyozyten exprimiert wird. Durch bioinformatorische Analysen identifizierten wir drei microRNAs (miR-200b-3p, miR-200c-3p, miR-429-3p), die putative Bindestellen in der 3'UTR der ACE2-mRNA besitzen und diese somit potenziell inhibieren können. Um dies zu testen, behandelten wir sowohl primäre Kardiomyozyten aus Rattenherzen als auch humane iPSC-abgeleitete Kardiomyozyten mit synthetischen microRNAs. Dabei stellte sich miR-200c-3p als potentester Inhibitor der ACE2-mRNA- und Proteinexpression heraus. In unabhängigen Experimenten validierten wir mit einem Luciferase-Reporter-Assay die spezifische Bindung zwischen miR-200c-3p und der ACE2 3'UTR [6]. Weiterführende Studien mit nationalen und internationalen Partnern laufen derzeit.

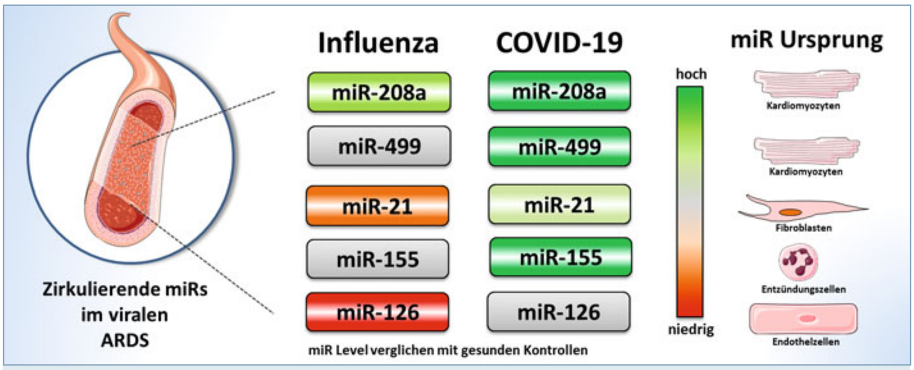

▲ **Abb. 2:** Plasmaspiegel von microRNAs (miRs) können zwischen Influenza- und COVID-19-Infizierten differenzieren, die an einem schweren Atemnotsyndrom (*acute respiratory distress syndrome*, ARDS) leiden. Die verschiedenen microRNAs sind mit unterschiedlichen Zelltypen assoziiert (Kardiomyozyten, Fibroblasten, Entzündungszellen, Endothelzellen), sodass die deregulierten microRNAs auf unterschiedliche Organbeteiligung hinweisen. Entnommen, adaptiert und ins Deutsche übersetzt aus [8], CC BY-NC 4.0.

## MicroRNA als prognostische Biomarker bei COVID-19

Zusätzlich zur Erforschung der microRNAs als therapeutisches Ziel, sind diese auch Gegenstand zahlreicher Biomarkerstudien. MicroRNAs können prinzipiell in allen Körperflüssigkeiten detektiert werden. Vor allem Blut- bzw. Plasmabiopsien eignen sich zur Bestimmung von microRNA-Spiegeln, da diese hier größtenteils gebunden an Proteine wie HDL und Argonaut oder verpackt in extrazelluläre Vesikel (EVs) vorliegen, was sie vor RNase-vermittelter Degradation schützt und ihnen Stabilität verleiht. Da microRNAs zudem häufig gewebe- und zelltypspezifisch exprimiert sind, sind sie vielversprechende Biomarker für verschiedene Konditionen, wie z. B. Leber- und Herztoxizität, sowie für eine ganze Reihe an Erkrankungen, wie z. B. Krebs, Herz-Kreislauf-Erkrankungen und Infektionskrankheiten (**Abb. 1**, [7]).

In unserer aktuellen Studie haben wir, basierend auf den zahlreichen kardiovaskulären Manifestationen von COVID-19, einen definierten Satz an kardiovaskulär- und entzündungsassoziierten microRNAs (miR-21, miR-126, miR-155, miR-208a und miR-499) in COVID-19-Patienten mit einem schweren Verlauf untersucht [8]. Während der ersten Corona-Welle in Deutschland verglichen wir zunächst in einer explorativen Kohorte die microRNA-Spiegel von schwerstkranken, mechanisch-beatmeten Patienten mit gesunden Kontrollprobanden. Die Spiegel der fünf rational ausgewählten microRNAs waren bei schwer an COVID-19 Erkrankten tatsächlich signifikant verändert. Das weist auf eine Assoziation von COVID-19 mit kardiovaskulärer Beteiligung hin. Aufgrund der bis dato limitierten Kohortengrößen (15 Kontrollen vs. 18 COVID-19), die zudem einen relativ großen Unterschied im Altersdurchschnitt aufwiesen, rekrutierten wir mit dem Aufkeimen der zweiten Corona-Welle weitere COVID-19-Patienten sowie altersangeglichene Kontrollprobanden. Darüber hinaus rekrutierten wir zusätzlich schwerkranke Influenzapatienten, die ebenfalls mechanisch beatmet werden mussten. Die Analyse der Validierungskohorten ergab, dass in COVID-19-Patienten die Spiegel entzündungsfördernder microRNAs, wie miR-155, und Kardiomyozyten-spezifischer microRNAs, wie miR-208a und miR-499, bei COVID-19-Patienten signifikant erhöht waren. In Übereinstimmung mit dem klinischen Bild der Erkrankung entsprach dies unseren Erwartungen. Obwohl sich im akuten klinischen Erscheinungsbild im Rahmen eines schweren ARDS COVID-19 und Influenza nur wenig unterscheiden, zeigten sich interessanterweise die Expressionslevel von miR-155 und miR-499 in COVID-19 auch signifikant gegenüber der Influenzagruppe erhöht. Zudem beobachteten wir, dass die Spiegel der Fibrose-assoziierten miR-21 bei COVID-19 erhöht waren – sowohl im Vergleich zu gesunden Kontrollen als auch zu Influenzapatienten (**Abb. 2**). Unsere Ergebnisse liefern somit einen weiteren direkten Anhaltspunkt für die myokardiale Beteiligung bei einer SARS-CoV-2 Infektion, wohingegen Influenza hauptsächlich eine pulmonale Reaktion auslöst. Unsere Ergebnisse lassen vermuten, dass die Plasmaspiegel von miR-21, miR-155, miR-208a und miR-499 ein Prädiktor für chronische Herzmuskelschäden und Entzündungen nach überstandener COVID-19-Erkrankung mit schwerem Verlauf sein könnten. Schätzungen zufolge leiden zirka zehn Prozent der COVID-19-Genesenen an Langzeitfolgen, wie Müdigkeit, Luftnot und auch Herzbeschwerden. Dieses bisher kaum verstandene und noch nicht klar abgegrenzte Krankheitsbild erhielt kürzlich den Namen Long-COVID [9].

Dies unterstreicht die Notwendigkeit für leicht zugängliche Biomarker, wie z. B. microRNAs aus Flüssigbiopsien, die prädiktive Informationen über potenzielle kardiovaskuläre Langzeitfolgen liefern können. Die Ergebnisse unserer Studie dienen als Grundlage für derzeitige breiter angelegte Studien, die den prognostischen Wert der microRNA-Messungen für den Langzeitverlauf von COVID-19-Patienten und von Long-COVID untersuchen. ■

## AUTOREN

### Thomas Thum

Hier steht
eine Anzeige.
Springer


Funding note

Open Access funding enabled and organized by Projekt DEAL.

Thomas Thum, Anselm A. Derda und Christian Bär (v. l. n. r.)

Christian Bär 2000–2006 Diplomstudium Biologie. 2007-2011 PhD in Genetics, University of Leicester, UK. 2012–2015 Postdoc in der Gruppe von Dr. M. Blasco am Spanischen Krebsforschungszentrum (CNIO) in Madrid. Seit 2015 Arbeitsgruppenleiter am Institut für Molekulare und Translationale Therapiestrategien der Medizinische Hochschule Hannover. 2021 Habilitation im Fach Experimentelle und Regenerative Kardiologie.

Anselm A. Derda 2010–2017 Studium Humanmedizin, Medizinische Hochschule Hannover (MHH). 2014–2015 Forschungsaufenthalt, National Heart & Lung Institute Imperial College London, UK. Seit 2017 Assistenzarzt, Klinik für Kardiologie und Angiologie, MHH. 2018 Doktor der Medizin, Institut für Molekulare und Translationale Therapiestrategien, MHH. Seit 2020 Förderung durch PRACTIS — Clinician Scientist Programm der MHH, gefördert durch die DFG.

Thomas Thum Studium Humanmedizin. 2004–2009 Facharztausbildung Innere Medizin und Kardiologie, Universität Würzburg. 2004–2009 PhD am National Heart and Lung Institut, Imperial College London, UK. Seit 2009 W3-Professor und Direktor Institut für Molekulare und Translationale Therapiestrategien. Seit 2013 Gastprofessur am National Heart and Lung Institut, Imperial College London. Seit 2021 Institutsleiter und Direktor am Fraunhofer ITEM, Hannover.


Literatur

[1] Puntmann VO Carerj ML Wieters I Outcomes of cardiovascular magnetic resonance imaging in patients recently recovered from coronavirus disease 2019 (COVID-19) JAMA Cardiol 2020 5 1265 1273 10.1001/jamacardio.2020.3557 32730619 PMC7385689
[2] Groß S Jahn C Cushman S SARS-CoV-2 receptor ACE2-dependent implications on the cardiovascular system: from basic science to clinical implications J Mol Cell Cardiol 2020 144 47 53 10.1016/j.yjmcc.2020.04.031 32360703 PMC7191280
[3] Viereck J Thum T Circulating noncoding RNAs as biomarkers of cardiovascular disease and injury Circ Res 2017 120 381 399 10.1161/CIRCRESAHA.116.308434 28104771
[4] Batkai S Genschel C Viereck J CDR132L improves systolic and diastolic function in a large animal model of chronic heart failure Eur Heart J 2021 42 192 201 10.1093/eurheartj/ehaa791 33089304 PMC7813625
[5] Täubel J Hauke W Rump S Novel antisense therapy targeting microRNA-132 in patients with heart failure: Results of a first-in-human Phase 1b randomized, double-blind, placebo-controlled study Eur Heart J 2021 42 178 188 10.1093/eurheartj/ehaa898 33245749 PMC7954267
[6] Lu D Chatterjee S Xiao K MicroRNAs targeting the SARS-CoV-2 entry receptor ACE2 in cardiomyocytes J Mol Cell Cardiol 2020 148 46 49 10.1016/j.yjmcc.2020.08.017 32891636 PMC7470794
[7] Gonzalo-Calvo D De, Vea A, Bär C et al. Circulating non-coding RNAs in biomarker-guided cardiovascular therapy: A novel tool for personalized medicine? Eur Heart J 40: 1643–165010.1093/eurheartj/ehy234 PMC6528150 29688487
[8] Garg A Seeliger B Derda AA Circulating cardiovascular microRNAs in critically ill COVID-19 patients Eur J Heart Fail 2021 23 468 475 10.1002/ejhf.2096 33421274 PMC8014268
[9] The L Facing up to long COVID Lancet 2020 396 1861 10.1016/S0140-6736(20)32662-3 33308453 PMC7834723
